# Enriched environment remodels the central immune environment and improves the prognosis of acute ischemic stroke in elderly mice with chronic ischemia

**DOI:** 10.3389/fimmu.2023.1114596

**Published:** 2023-03-09

**Authors:** Shehong Zhang, Yonggang Zhang, He Liu, Fengfeng Wu, Zhihong Wang, Liqin Li, Huilian Huang, Sheng Qiu, Yuntao Li

**Affiliations:** ^1^ Department of Rehabilitation Medicine, Department of Neurosurgery, Huzhou Central Hospital, Affiliated Huzhou Hospital, Zhejiang University School of Medicine, Huzhou, China; ^2^ Huzhou Key Laboratory of Basic Research and Clinical Translation for Neuromodulation, Huzhou, China; ^3^ Department of Neurosurgery, Huzhou Central Hospital, Affiliated Huzhou Hospital, Zhejiang University School of Medicine, Huzhou, China

**Keywords:** enriched environment, chronic cerebral hypoperfusion, ischemic stroke, neuroinflammation, elderly mice, cognitive function

## Abstract

With the aging of many populations, cognitive and motor dysfunction caused by ischemic stroke (IS) secondary to long-term chronic cerebral ischemia presents a global problem. Enriched environment (EE), a classic paradigm of environment response and genetic interaction, has shown tremendous influence on the brain. This research aimed to investigate the potential effect of EE on cognitive and motor function in mice with chronic cerebral ischemia and secondary IS. In the chronic cerebral hypoperfusion (CCH) phase, EE treatment improved behavior performance by alleviating neuronal loss and white matter myelin damage, promoting the expression of brain-derived neurotrophic factor (BDNF) and phosphor-cAMP response element binding protein (p-CREB). Furthermore, infiltration of microglia/macrophages and astrocytes was inhibited, and the levels of IL-1β and TNFα were decreased. In the IS phase, EE altered the neuronal outcome on day 21 but not on day one after IS. In addition, EE inhibited IS-induced infiltration of microglia/macrophages and astrocytes, mediated the polarization of microglia/macrophages, and reduced pro-inflammatory factors. Importantly, EE improved IS-induced cognitive and motor deficits on day 21. Collectively, our work demonstrates that EE protects mice from cognitive and motor dysfunction and inhibits neuroinflammation caused by CCH and IS.

## Introduction

Stroke remains the second leading cause of death in the world, and the elderly constitute the majority of stroke patients (1). Unfortunately, elderly patients with ischemic stroke are often excluded from clinical trials due to multiple underlying diseases and a wide variation in therapeutic effects, leading to slow progress in the treatment of elderly patients with ischemic stroke (1).

Elderly patients with ischemic stroke often suffer from a long history of chronic cerebral hypoperfusion (CCH). CCH is one of the pathophysiological mechanisms contributing to intellectual decline, including global cognitive performance, executive function, and processing speed in aging and vascular cognitive impairment (2, 3). Animal models of chronic cerebral hypoperfusion have demonstrated a causal link between brain hypoperfusion, white matter damage, and cognitive deficits (4–6). An elevated inflammatory response and oxidative stress are implicated in the pathogenesis of white matter damage following cerebral hypoperfusion (4), spinal cord injury (7), and ischemic stroke (8), along with the activities of both astrocytes and microglia/macrophages (9), which also undergo inflammatory transitions after acute insults. Inflammation leads to oxidative stress, which is also proposed to contribute mechanistically to white matter structural damage. This in turn aggravates inflammation and forms a vicious circle (10).

An enriched environment (EE) has been shown to improve cognitive dysfunction after ischemic stroke, but the underlying mechanism needs further clarification (11). Studies based on exposure to EE seem to provide a promising low-cost strategy with no side effects. Furthermore, CCH alters neuroinflammatory homeostasis (12). Therefore, investigating the role of EE in neuroinflammation and the pathological processes of chronic cerebral hypoperfusion and secondary ischemic stroke in elderly patients is of great significance.

In this study, a chronic cerebral hypoperfusion model was constructed by unilateral common carotid artery occlusion (UCCAO) in elderly mice. On this basis, middle cerebral artery occlusion (MCAO) was performed to set up an acute cerebral ischemia model. Our data suggested that EE could improve the outcome of elderly mice with CCH and acute ischemic stroke by remodeling the central immune environment to alleviate the damage to neurons and white matter.

## Methods

### Animals

36-week-old C57BL/6 male mice were purchased from the Shanghai Laboratory Animal Center (SLAC) and were acclimated to the new housing environment for 7 days. A reversed 12:12-hour light/dark cycle was set and the mice were allowed free access to water and standard food. All behavioral tests were performed between 9 and 12 am, as designated by the experiment. All of the animal protocols were approved by the Huzhou Central Hospital Animal Care and Use Committee and were in complete accordance with the National Institutes of Health Guide for the Care and Use of Laboratory Animals. Environmental enrichment housing conditions were established according to our previous work (13).

### Chronic cerebral hypoperfusion (CCH) model

The CCH model was induced by left unilateral common carotid artery (UCCA) occlusion surgery, according to the previously published literature (6). Briefly, experimental mice were deeply anesthetized using 2% isoflurane in 30% O_2_, then placed in the supine position. The left UCCA was exposed and a silk thread was used to ligate and permanently block the UCCA. Throughout the surgery, the body temperature was maintained at around 37 °C. The sham group was subjected to the same operation without carotid occlusion.

### Ischemic stroke (IS) model

The IS model was established as previously described (14). In short, mice were anesthetized and the body temperature was maintained as described in the CCH model. Subsequently, the left middle cerebral artery was ligated with a suture (Doccol, Corp, Redlands) for 1 hour to induce IS. Sham-operated mice were subjected to the same procedure but without ligation.

### Animal behavioral analysis

#### Novel object recognition (NOR) test

Recognition memory ability was evaluated by the NOR test, as previously described (6). The discrimination index ratio was calculated as follows: time novel object/(time novel object - time familiar object). A ratio > 0.5 indicated the exploration of more than two objects, a higher exploratory time for the novel object, and a preference for the familiar object.

#### Open field test (OFT)

The anxiety and general ambulatory ability of animals were evaluated by using this test, which was performed as previously described (4). The parameters evaluated included the time spent in the different zones, latency to the different zones, and the distance of movement.

#### Y maze test

This test was also conducted as previously described (15). The behavior of the mice during free exploration was observed for 6 minutes.

### Neurological evaluation and infarct volume measurement

On day 1 and day 21 after MCAO, the neurological deficit score of the mice was assessed, with scores ranging from 0 (without observable neurological deficit) to 4 (no spontaneous motor activity and loss of consciousness). Then, the mice were sacrificed and the brains were collected, which were sliced into four coronal sections of 2 mm thickness. The sections were stained by 1.5% 2,3,5-triphenyltetrazolium chloride (TTC) staining solution. After staining, the brain sections were scanned and the infarct volume was evaluated by Image J software as previously described (14). The researcher was blinded to the intervention groups.

### Immunofluorescence staining

Mice were anesthetized with an isoflurane overdose in 70% N_2_O and 30% O_2_ and perfused with 0.9% NaCl and 4% paraformaldehyde (PFA), after which the brains were collected. The brains were placed into a vessel with a flat bottom to avoid brain tissue deformation and fixed in 4% PFA overnight, and then were deposited in 30% sucrose at 4°C. The brain tissues were sectioned using a freezing microtome and were blocked and incubated with the primary antibodies at 4°C overnight. The primary antibodies are listed in **Table 1**. After washing, the samples were incubated for 1 hour at room temperature with suitable secondary antibodies. Finally, the nuclei were stained with DAPI (4,6-diamino-2-phenylindole, 1:1000) for 20 minutes at room temperature. Fluorescence images were observed and captured using an epifluorescence microscope (Olympus Optical, Japan).

**Table 1:** Primary antibodies used in this study.

Reagents	Source	Catalog number	Application details
NeuN	abcam	ab104224	IB 1:1000; IF 1:200
MBP	Cell Signaling Technology	78896	IB 1:800; IF 1:200
GFAP	Cell Signaling Technology	80788	IB 1:1000; IF 1:200
IBA1	Cell Signaling Technology	17198	IB 1:1000; IF 1:200
iNOS	Cell Signaling Technology	13120	IB 1:1000
ARG-1	Cell Signaling Technology	93668	IB 1:1000
BDNF	abcam	ab108319	IB 1:1000
p-CREB	abcam	ab32096	IB 1:1000
CREB	abcam	ab178322	IB 1:1000
GAPDH	Cell Signaling Technology	5174	IB 1:2000
β-ACTIN	Cell Signaling Technology	3700	IB 1:1000

### Western blot

Mice were sacrificed with an isoflurane overdose and the brains were harvested. The brain tissues of mice were carefully separated at specific time points. Western blot was performed according to standard protocol with proper antibodies, as previously reported (16). The primary antibodies are displayed in **Table 1**.

### Transmission electron microscopy

The brains were collected as described above. The right corpus callosum was isolated and cut into approximately 1 mm (3) brain pieces, and then placed in 2.5% glutaraldehyde and 4% PFA to post-fix at 4°C for 2 hours. After washing, the brains were fixed in 1% osmium tetroxide for 35 minutes. The specimens were dehydrated using different gradient alcohol and embedded. After dehydration and embedment, serial ultrathin sections (80 nm) were prepared, stained with lead citrate and uranyl acetate, and observed and imaged with a JEM-2100 transmission electron microscope (Tokyo, Japan). The ratio of demyelinated axons in the total axons (%) was calculated.

### Statistics

All the statistical analyses were performed with GraphPad Prism software version 9 (GraphPad Software, Inc., San Diego, CA). All the data were analyzed by using one-way ANOVA followed by the Tukey test for multiple comparisons. *P* < 0.05 was considered statistically significant.

## Results

### Effect of EE on behavior analysis in CCH mice

**Figure 1:**
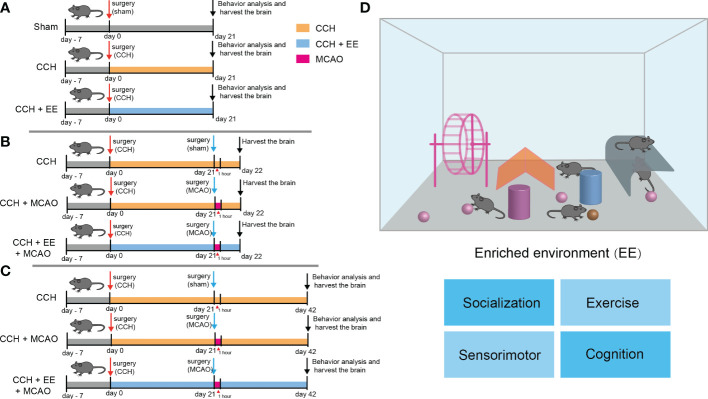
The design of the experiment and schematic of enriched environment **(A–C)** The design of the experiment. **(A)** Male C57 mice were subjected to CCH surgery on the first day, and CCH mice were partly given general treatment and partly given EE treatment. The sham group was given general treatment after sham operation. Behavioral analysis was performed and brain tissue was collected on day 21. **(B)** CCH mice with or without EE treatment were subjected to MCAO on day 21, and brain tissue was collected on day 22. **(C)** CCH mice with or without EE treatment were subjected to MCAO on day 21. Behavioral analysis was performed and brain tissue was collected on day 42. **(D)** A schematic of enriched environment.

**Figure 2:**
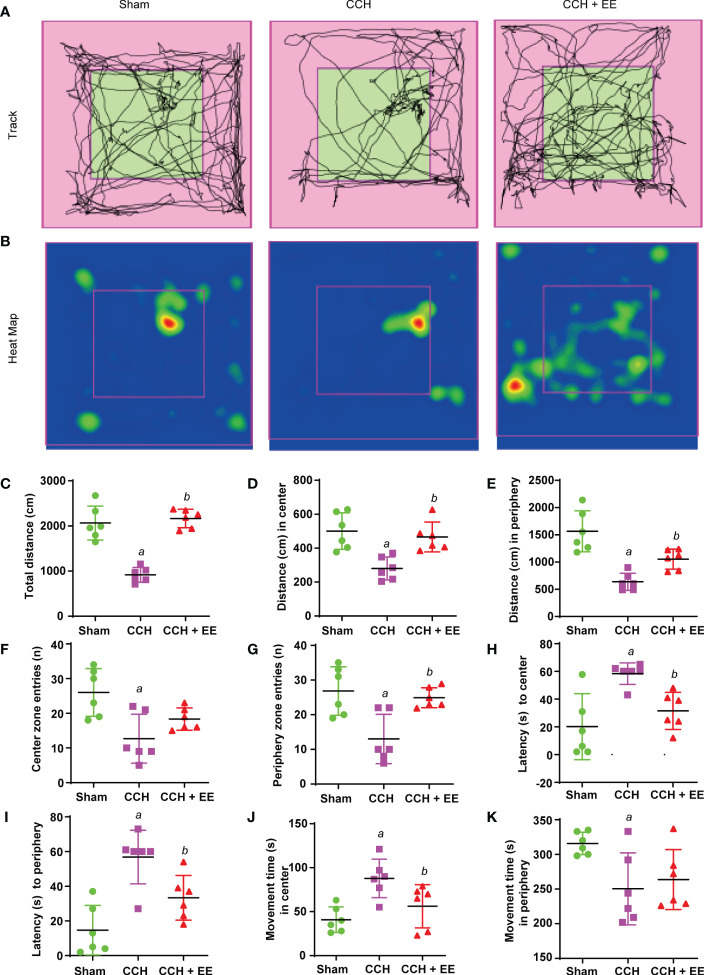
Effect of EE treatment on open field test in CCH mice. **(A)** Movement track of mice. **(B)** Heat map of mouse movement. **(C-K)** Behavior analysis of open field test, including total distance **(C)**, distance in center **(D)**, distance in periphery **(E)**, center zone entries **(F)**, periphery zone entries **(G)**, latency to center **(H)**, latency to periphery **(I)**, movement time in center **(J)** and movement time in periphery **(K)**. Values represent the mean ± s.d. *^a^P* < 0.05 vs. Sham; *^b^P* < 0.05 vs. CCH group (n = 6).

Since studies have shown that EE could improve cognitive impairment in neurodegenerative diseases, including Huntington’s disease and Alzheimer’s disease (17), behavior analysis was performed to investigate the effect of EE treatment in CCH mice. As illustrated in **Figure 1A**, the behavior analysis was performed on mice 21 days after CCH, including the OFT, Y maze test, and NOR test. The movement track and heatmap of the OFT are shown in **Figures 2A, B**. The results revealed decreased movement distance and entry times compared with the sham group, and latency was increased both in the center and the periphery (**Figures 2C–I**). However, the time spent in the center was greater than in the periphery (**Figures 2J, K**). Interestingly, EE treatment partly reversed most changes, except for center zone entries and movement time in the peripheral regions (**Figures 2F, K**). In the Y maze test, a lower total number of arm entries and alternation rates were observed in CCH mice compared with the sham group (**Supplementary Figures S1A–D**). However, EE treatment partly improved this result. In the NOR test, EE treatment significantly improved the discrimination index compared with the CCH group (**Supplementary Figure S1E**). Collectively, the results indicated that CCH mice suffered an obvious cognitive impairment and EE treatment could improve the outcome.

### The effect of EE on hippocampus and corpus callosum in CCH mice

**Figure 3:**
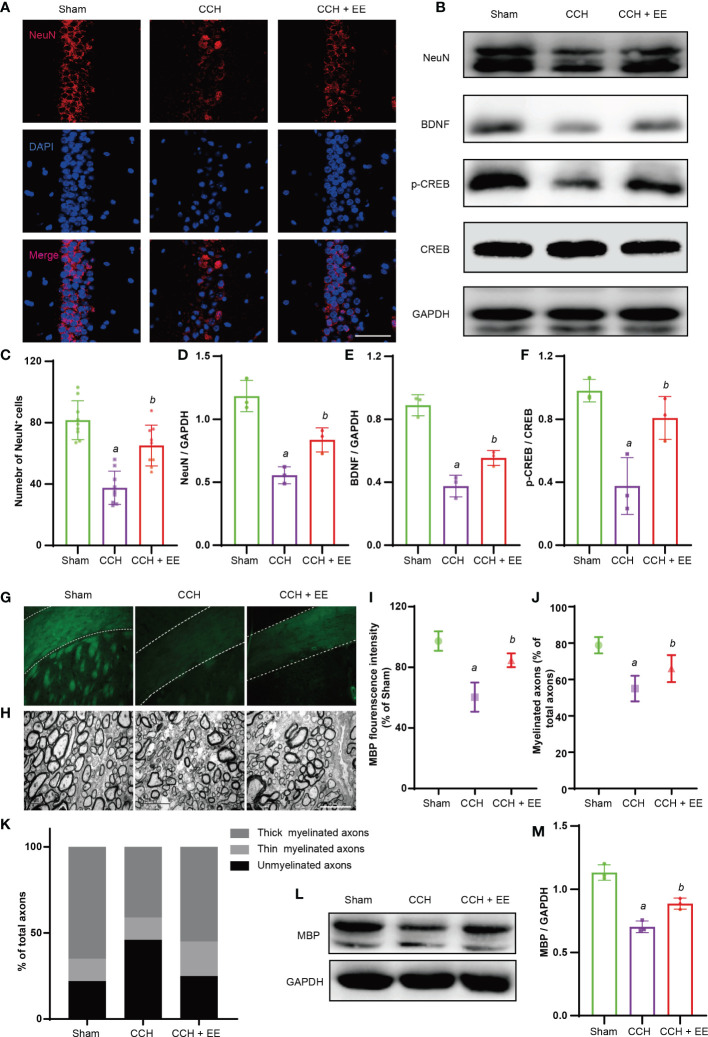
Effect of EE on hippocampus and corpus callosum in CCH mice. **(A, C)** Immunofluorescence staining of NeuN in hippocampus (n = 6), scale bar = 40 μm. **(B, D–F)** Western blot analysis of NeuN, BDNF, P-CREB, and CREB in hippocampus, (n = 3). **(G, I)** Immunofluorescence staining of MBP in corpus callosum, (n = 3), scale bar = 50 μm. **(H, J, K)** The result of electron microscopy, scale bar = 5 μm. (n = 3). **(L, M)** Western blot analysis of MBP in corpus callosum (n = 3). Values represent the mean ± s.d. *^a^P* < 0.05 vs. Sham; *^b^P* < 0.05 vs. CCH group.

Hippocampal neuron loss and white matter damage after chronic cerebral ischemia are important causes of cognitive dysfunction. Hence, the damage to hippocampal neurons and the corpus callosum was determined. Immunofluorescence staining showed a significantly lower number of NeuN-positive cells (neuronal markers) in the hippocampus region after chronic ischemia, indicating a loss of neurons (**Figures 3A, C**). Moreover, a lower fluorescence intensity of myelin basic protein (MBP) was observed in the corpus callosum, indicating myelin sheath damage (**Figures 3G, I**). Consistently, EE treatment partly rescued the outcome of hippocampal neurons and improved the fluorescence intensity of MBP (**Figures 3A, C, G, I**). Meanwhile, electron microscopy was performed to observe the myelin ultrastructure of the corpus callosum, revealing a reduced percentage of myelinated axons in CCH mice compared with the sham group (**Figures 3H, J**). As decribed (6), we evaluated the proportion of thick myelinated axons, thin myelinated axons, and unmyelinated axons. The results showed that the percentage of thick myelinated axons were decreased, thin myelinated axons had no significant changes, but unmyelinated axons were increased after CCH (**Figures 3H, K**). However, EE treatment increased the percentage of thick and thin myelinated axons and decreased the percentage of unmyelinated axons. Furthermore, western blot analysis was performed for NeuN, brain-derived neurotrophic factor (BDNF) and cAMP response element binding protein (CREB), and phosphor-CREB (p-CREB) in the hippocampus, demonstrating that EE rescued the downregulation of NeuN (**Figures 3B, D**). Interestingly, BDNF and CREB are known as proteins related to neuronal growth and differentiation, and EE upregulated the levels of BDNF and p-CREB (**Figures 3B, E, F**). Subsequently, the levels of MBP in the corpus callosum were evaluated. EE upregulated the MBP levels, which was similar to the immunofluorescence staining results (**Figures 3L, M**). Therefore, our results indicated that EE treatment protected the hippocampus and corpus callosum from CCH injury.

### The effect of EE on neuroinflammation in CCH mice

**Figure 4:**
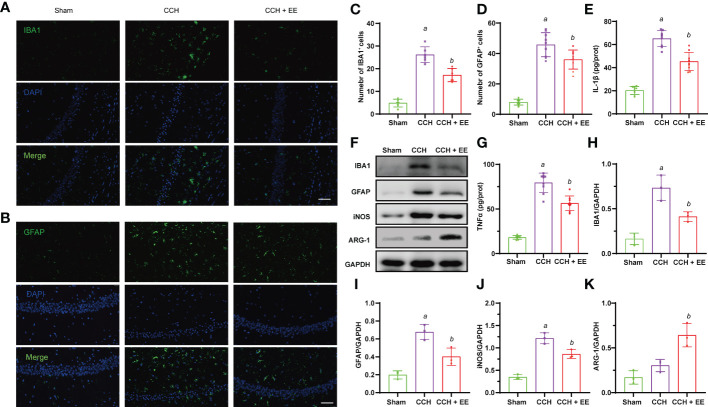
Effect of EE on neuroinflammation in CCH mice. **(A, C)** Immunofluorescence staining of IBA1 in hippocampus region (n = 9). **(B, D)** Immunofluorescence staining of GFAP in hippocampus region (n = 9), scale bar = 50 μm. **(E, G)** The levels of IL-1β and TNFα in hippocampus tissue (n = 9). **(F, H–K)** Western blot analysis of IBA1, GFAP, iNOS, and ARG-1 in hippocampus tissue (n = 3). Values represent the mean ± s.d. *^a^P* < 0.05 vs. Sham; *^b^P* < 0.05 vs. CCH group.

Given the potential effects of EE on inflammation and the important role of inflammation in CCH, we investigated whether EE could inhibit neuroinflammation in CCH mice. An obvious increase in IBA1-positive cells (microglia/macrophages marker) and GFAP-positive cells (astrocytes marker) was observed in the ischemic region (**Figures 4A–D**), indicating microglia/macrophage and astrocyte infiltration into the ischemic region after CCH. But EE partly reversed their changes. Western blot analysis of IBA1 and GFAP showed the same trend, and EE treatment downregulated IBA1 and GFAP (**Figures 4F, H, I**). The different polarization states of microglia/macrophages lead to different functions, and it is generally believed that M1-like polarization promotes inflammation and aggravates tissue damage, while M2-like polarization inhibits inflammation and promotes tissue repair (18). Western blot analysis showed that EE downregulated the expression of iNOS, a marker of M1-like microglia/macrophages, and upregulated the levels of ARG-1, a marker of M2-like microglia/macrophages. The results indicated that EE might regulate the polarization of microglia/macrophages (**Figures 4F, J, K**). Moreover, the levels of IL-1β and TNFα in the brain were assessed by ELISA, revealing increased levels after CCH, while EE alleviated their levels (**Figures 4E, G**). Collectively, our data indicated that EE inhibited the neuroinflammation induced by CCH.

### The effect of EE on CCH mice one day after MCAO

**Figure 5:**
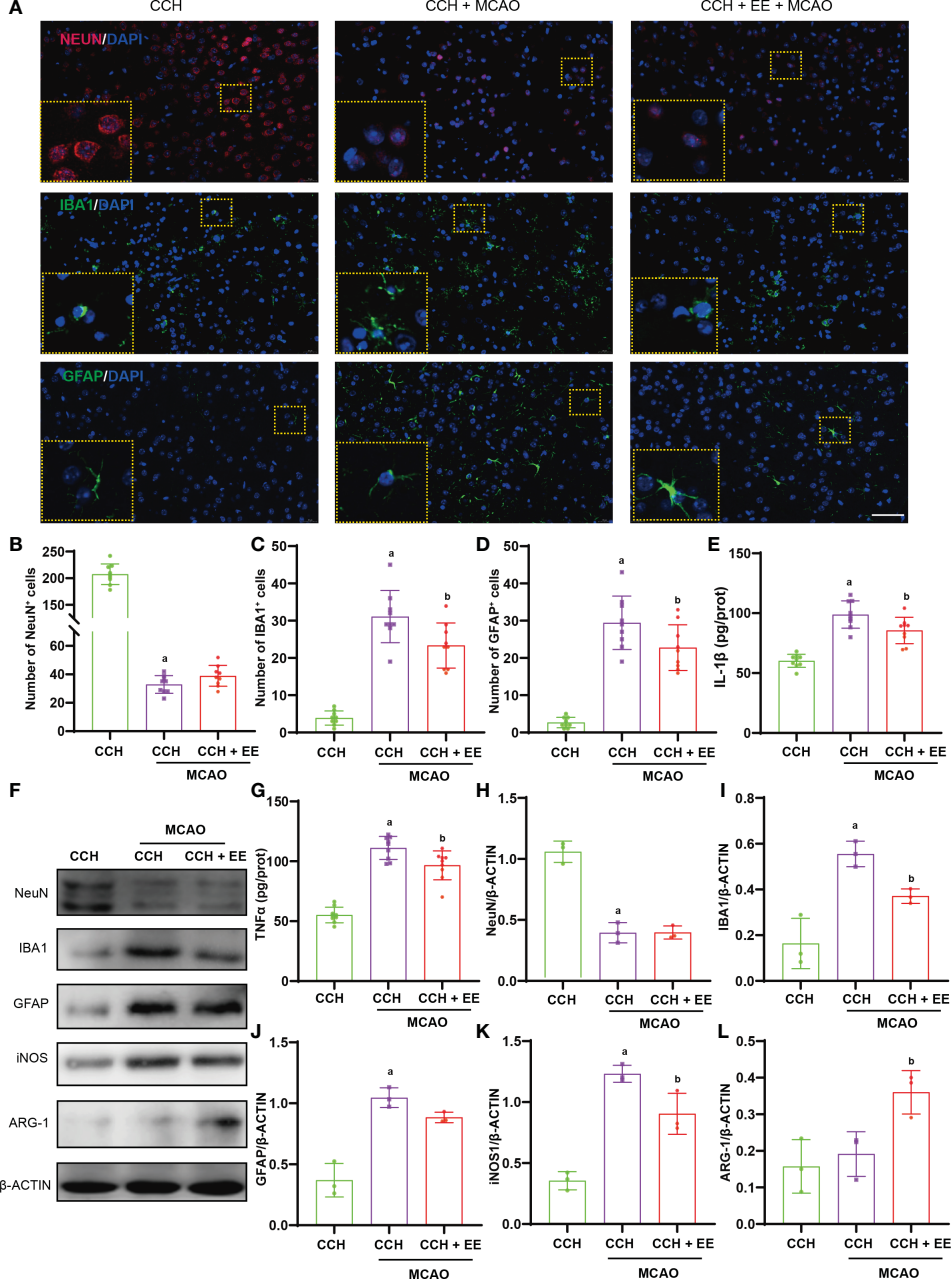
Effect on EE on CCH mice one day after MCAO. **(A)** Immunofluorescence staining of NeuN (up), IBA1 (mid), and GFAP (down) in penumbra region (n=9), scale bar = 50 μm. **(B–D)** Quantification results of NeuN-positive, IBA1-positive, and GFAP-positive cells in penumbra region. **(E, G)** The levels of IL-1β and TNFα in penumbra region (n = 9). **(F, H–L)** Western blot analysis of NeuN, IBA1, GFAP, iNOS, and ARG-1 in penumbra region (n = 3). Values represent the mean ± s.d. *^a^P* < 0.05 vs. CCH group; *^b^P* < 0.05 vs. CCH with MCAO group.

Chronic cerebral ischemia is a risk factor for stroke, and many elderly patients often experience chronic ischemic events before a stroke. As shown in **Figure 1B**, middle cerebral artery occlusion (MCAO) was performed in CCH mice to simulate ischemic stroke after CCH, and the role of EE was investigated. First, the effect of EE on stroke outcome was assessed one day after MCAO. As shown in **Supplementary Figures S2A, B**, TTC staining demonstrated that although EE could reduce the cerebral infarct volume to a certain extent, it was not statistically significant (**Supplementary Figure S2C**). Furthermore, no significant difference was observed in neurological deficit scores. Immunofluorescence staining showed that NeuN-positive cells were significantly reduced, and IBA1-positive and GFAP-positive cells were increased in the penumbra region one day post-MCAO (**Figures 5A–D**). These findings were consistent with the western blot analysis of IBA1 and GFAP (**Figures 5F, I, J**), indicating loss of neurons and infiltration of microglia/macrophages and astrocytes in the penumbra region. In the EE treatment group, the expression of IBA1 was downregulated, and microglial/macrophage and astrocyte infiltration were reduced. In addition, compared with the CCH group, iNOS was upregulated in the penumbra region, while ARG-1 showed no significant difference one day after MCAO. EE treatment downregulated iNOS and upregulated ARG-1 (**Figures 5F, K, L**). Meanwhile, the levels of IL-1β and TNFα were further increased after MCAO, while they were decreased after EE treatment (**Figures 5E, G**). However, EE treatment did not increase the number of NeuN-positive cells and did not upregulate the expression of NeuN in the penumbra region one day after MCAO (**Figures 5A, B, F, H**). Collectively, these data indicated that EE could alleviate neuroinflammation, but could not significantly improve the outcome of CCH mice one day after MCAO.

### The effect of EE on behavior analysis in CCH mice 21 days after MCAO

**Figure 6:**
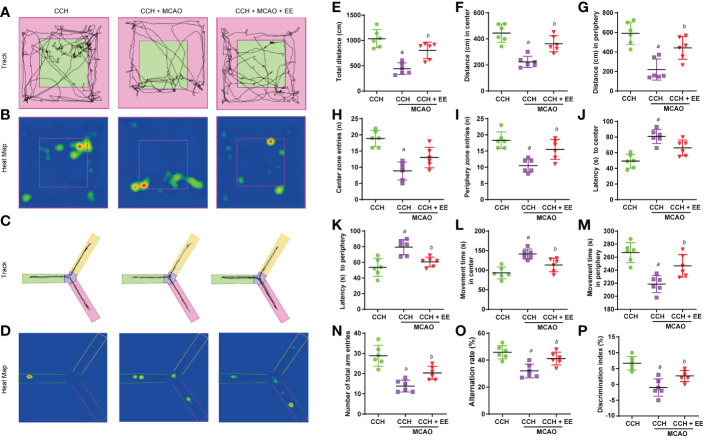
Results of behavior analysis in CCH mice 21 days after MCAO. **(A)** Movement track of mice in open field test. **(B)** Heat map of mouse movement in open field test. **(C)** Movement track of mice in Y maze test. **(D)** Heat map of mouse movement in Y maze test. **(E–M)** Behavior analysis of open field test, including total distance **(E)**, distance in center **(F)**, distance in periphery **(G)**, center zone entries **(H)**, periphery zone entries **(I)**, latency to center **(G)**, latency to periphery **(K)**, movement time in center **(L)** and movement time in periphery **(M)**. **(N, O)** Behavior analysis of Y maze test. **(P)**. Values represent the mean ± s.d. *^a^P* < 0.05 vs. CCH group; *^b^P* < 0.05 vs. CCH with MCAO group (n = 6).

Given the results after one day, the effect of EE on CCH mice at 21 days after MCAO was further investigated. Although the TTC staining results showed that EE treatment still could not reduce the cerebral infarct volume in CCH mice, the neurological deficit score was effectively reduced (**Supplementary Figures S3A–C**). Then, as shown in **Figure 1C**, a behavior analysis on day 42 was performed. The results of the OFT (**Figures 6A, B, E–M**), Y maze (**Figures 6C, D, N, O**), and NOR tests (**Figure 6P**) indicated aggravation of the motor and cognitive impairment of CCH mice after MCAO (**Figures 6A–P**). A lower movement distance and the number of entries were observed (**Figures 6E–I**), while latency time and time spent in the center were increased in OFT (**Figures 6J–M**). The total number of arm entries and alternation rates were reduced in the Y maze (**Figures 6N, O**), and the discrimination index was decreased in the NOR test (**Figure 6P**). However, EE treatment partly reversed most of their changes, except for center zone entries (**Figure 6H**) and latency time at the center (**Figure 6J**), suggesting that EE could improve motor and cognitive impairment 21 days after MCAO.

### The effect of EE on neurons in CCH mice 21 days after MCAO

**Figure 7:**
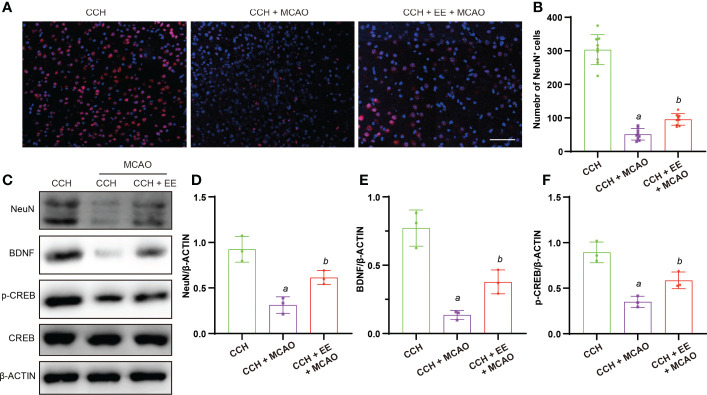
Effect of EE on neurons in CCH mice 21 days after MCAO. **(A, B)** Immunofluorescence staining of NeuN and quantification result of NeuN-positive cells in penumbra region (n = 9), scale bar = 50 μm. **(C–F)** Western blot analysis of NeuN, BDNF, p-CREB, and CREB in penumbra region (n = 3). Values represent the mean ± s.d. *^a^P* < 0.05 vs. CCH group; *^b^P* < 0.05 vs. CCH with MCAO group.

In addition to the effect of EE on behaviors at 21 days after MCAO, we investigated the outcome of neurons in mice. As shown in **Figures 7A, B**, a greater number of NeuN-positive cells was observed after EE treatment than without EE treatment. The western blot analysis of NeuN in the penumbra region showed a similar trend (**Figures 7C, D**). Furthermore, the levels of BDNF and p-CREB were downregulated after MCAO, which was rescued by EE treatment (**Figures 7C, E, F**). These results suggest that EE treatment improved neuronal outcomes and the levels of cognitive-related proteins 21 days after MCAO.

### The effect of EE on neuroinflammation in CCH mice 21 days after MCAO

**Figure 8:**
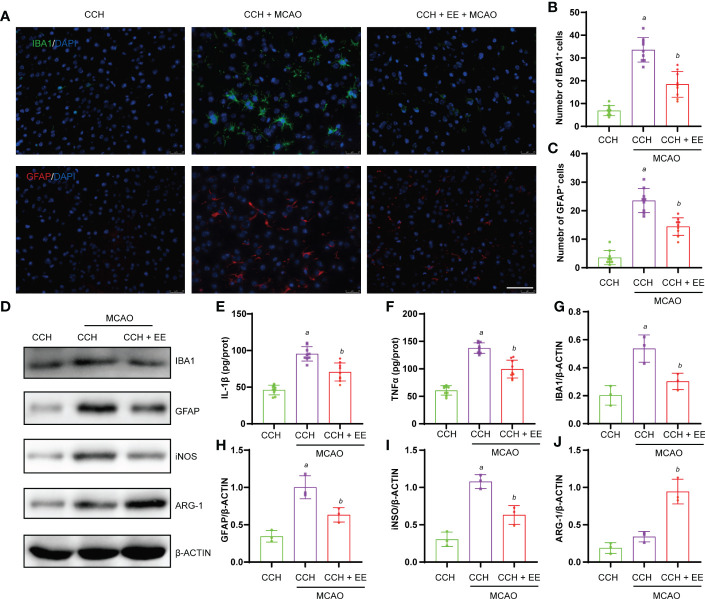
Effect of EE on neuroinflammation in CCH mice 21 days after MCAO. **(A, B)** Immunofluorescence staining of IBA1 and quantification result of IBA1-positive cells in penumbra region (n = 9). **(A, C)** Immunofluorescence staining of GFAP and quantification result of GFAP-positive cells in penumbra region (n = 9). **(E, F)** The levels of IL-1β and TNFα in penumbra region measured by ELISA (n = 9). **(D, G–J)** Western blot analysis of IBA1, GFAP, iNOS, and ARG-1 in penumbra region (n = 3). Scale bar = 50 μm. Values represent the mean ± s.d. *^a^P* < 0.05 vs. CCH group; *^b^P* < 0.05 vs. CCH with MCAO group.

Chronic inflammation after ischemic stroke is critical for cognitive function recovery. Therefore, inflammation in the brain tissue of CCH mice was assessed after 21 days of MCAO. The immunofluorescence staining (**Figures 8A–C**) and western blot results (**Figures 8D, G, H**) showed significant infiltration of microglia/macrophages and astrocytes into the penumbra 21 days after MCAO, which was attenuated by EE treatment. Importantly, EE treatment also regulated the polarization of microglia/macrophages and significantly downregulated iNOS and upregulated ARG-1 (**Figures 8D, I, J**). In addition, the treatment reduced the levels of IL-1β and TNFα (**Figures 8E, F**). Therefore, our results further indicated that EE alleviates the neuroinflammation of CCH mice 21 days after MCAO.

## Discussion

Cognitive and motor impairment caused by vascular dysfunction is a common problem faced by all countries in the world, which increases with aging populations (19–21). At present, a large number of studies have focused on the changes in cognitive and motor function after vascular dysfunction each year, including but not limited to chronic cerebral hypoperfusion and ischemic stroke. Despite the large number of solutions found, the clinical translation rate of these ideas remains extremely low (22). One of the reasons is the inappropriateness of the animal model. While cognitive deficits due to vascular dysfunction are often a disease of old age, many studies are based on young animals, which is equivalent to human adolescence (22–24). This is unreasonable because many functions of adolescent individuals are clearly different from those of old individuals, including nervous system functions (25, 26). In addition, although some drugs have entered the clinical trial stage and a few have been approved for use, the heterogeneity of drug efficacy, drug side effects, and the resulting economic burden remains a major problem (27, 28). Therefore, studies based on exposure to an enriched environment (EE) have been performed to improve the prognosis of vascular dementia and seem to provide a promising low-cost choice with no side effects.

The concept of the enriched environment is based on the continuous effect of individual practice on the function of the central nervous system and is considered a classic example to illustrate the crosstalk between genes and the environment (29). The schematic diagram of the enriched environment is shown in **Figure 1D**. Despite the diversity of the environment, it mainly includes four aspects: exercise, socialization, sensorimotor, and cognition (11, 29). Interestingly, their synergistic effects are more significant than their individual effects (30). The effect of environmental enrichment on the brain is remarkable. Initially, it was only incidentally found to improve animal performance in behavioral tests (29), but now has been widely reported to improve the prognosis of neurodegenerative diseases (11). In ischemic stroke, for example, although EE has rarely been reported to significantly reduce infarct volume (11, 31, 32), long-term EE treatment can promote hippocampal and cortical neuronal survival (33). Furthermore, since the formation of new synapses is an effective manifestation of individual learning and memory, EE can not only promote neuronal regeneration but also promote synaptic remodeling to improve cognitive dysfunction after stroke (34, 35). In addition, EE has enormous clinical value as a behavioral intervention to improve stroke outcomes. Clinical studies have reported that EE intervention can improve cognitive dysfunction in patients with multiple sclerosis and Parkinson’s disease, showing great potential (11). Moreover, EE has also been used in clinical research on stroke and achieved certain clinical effects. Although EE did not significantly improve the cognitive function scores of stroke patients, many studies have shown that EE intervention can significantly increase activity participation, which greatly improves the willingness of stroke patients to take part in activities (36). Interestingly, cognitive stimulation was superior to exercise stimulation in improving cognitive function in patients (37). In addition, a large number of patients often suffer from chronic hypoperfusion before a stroke, while clinical EE intervention is performed after the stroke. In animal models, studies have reported that pre-intervention with EE can reduce stroke injury (11), so we speculated whether EE intervention at the stage of chronic hypoperfusion would have a better effect. In this study, a CCH mouse model was constructed, revealing that EE could improve the cognitive and motor dysfunction of CCH mice. At the microscopic level, EE ameliorated hippocampal neuron loss and myelin damage and promoted the expression of cognitive-related proteins (BDNF, p-CREB). Furthermore, we constructed an MCAO model in CCH mice and found that EE improved the motor and cognitive functions of IS mice 21 days after MCAO, with an increase in the number of penumbral neurons and an upregulation of functionally related proteins BDNF and p-CREB.

The importance of inflammation in neurodegenerative diseases is self-evident. The phagocytosis of activated macrophages can aggravate tissue damage or promote tissue repair, depending on the activation state of macrophages (38). Macrophages in different states synthesize and secrete different types of inflammatory molecules with different outcomes. For example, IL-1β secreted by M1-like macrophages promotes inflammation and aggravates neuronal damage, while IL-4 produced by M2-like macrophages inhibits inflammation and promotes wound repair (39). Astrocytes play a similar role as they can promote nerve repair by producing trophic factors and are also able to recruit inflammatory cells to aggravate tissue damage (40). In the process of cerebral ischemia, excessive activation of microglia and astrocytes can cause damage to the cortex and white matter and affect behavioral function (39, 41). In contrast, activation by targeting microglia and astrocytes improves prognosis (18, 42). In this study, we found that EE inhibited the activation of microglia and astrocytes after ischemia, both during the chronic ischemic phase and after secondary IS. It also reduced the production of inflammatory factors. As mentioned above, microglial activation plays a dual role. Although the expression of the M1 marker iNOS and the M2 marker ARG-1 suggested that EE may regulate microglial polarization during ischemia, this evidence is not sufficient to reflect the state of the cells themselves. As M1 and M2 microglia are different states of the same cell, they can be transformed into each other (43). Moreover, although EE attenuated microglial and astrocyte activation, the neuronal outcome was not significantly improved in the acute phase after secondary acute stroke. On the one hand, the factors that affect neuronal outcome after stroke are complex, including but not limited to neuroinflammation. On the other hand, the status of inflammatory cells at this time is not clear, and studies have shown that the loss of microglia at the initial stage can increase the accumulation of neutrophils (44), which could disrupt the blood-brain barrier and aggravate neuronal damage (45).

In conclusion, our results suggest that EE can mitigate cognitive dysfunction in the chronic ischemic phase and ischemic stroke by improving neuronal outcomes and modulating neuroinflammation. Therefore, EE is of great significance in improving the prognosis of acute ischemic stroke in elderly mice with chronic ischemia.

## Data availability statement

The original contributions presented in the study are included in the article/**Supplementary Material**. Further inquiries can be directed to the corresponding authors.

## Ethics statement

The animal study was reviewed and approved by Laboratory Animal Management and Ethics Committee of Huzhou Central Hospital.

## Author contributions

SZ and YZ: Performing the experiment and writing the original draft. HL, FW and ZW: Performing the experiment. LL, HH: Data Curation. SQ and YL: Project administration. All authors listed have made a substantial, direct, and intellectual contribution to the work, and approved it for publication.

## Supplementary material

Supplementary Figure 1 Results of Y maze test and NOR test. **(A)** Movement track of mice. **(B)** Heat map of mouse movement. **(C, D)** Behavior analysis of Y maze test. **(E)** Behavior analysis of NOR test. Values represent the mean ± s.d. *^a^P* < 0.05 vs. Sham; *^b^P* < 0.05 vs. CCH group (n = 6).

Supplementary Figure 2 Results of TTC staining and neurological score one day after MCAO. **(A, B)** TTC staining of CCH mice one day after MCAO. **(C)** Neurological score of CCH mice one day after MCAO. Values represent the mean ± s.d. *ns* non significance. (n = 6).

Supplementary Figure 3 Results of TTC staining and neurological score 21 days after MCAO. **(A, B)** TTC staining of CCH mice 21 days after MCAO. **(C)** Neurological score of CCH mice 21 days after MCAO. Values represent the mean ± s.d. *ns* non significance. (n = 6).
