# Supplementary figures and images for: Enriched environment remodels the central immune environment and improves the prognosis of acute ischemic stroke in elderly mice with chronic ischemia
Source: Front Immunol. 2023 Mar 9;14:1114596. doi: 10.3389/fimmu.2023.1114596 (PMC10033834; doi:10.3389/fimmu.2023.1114596)

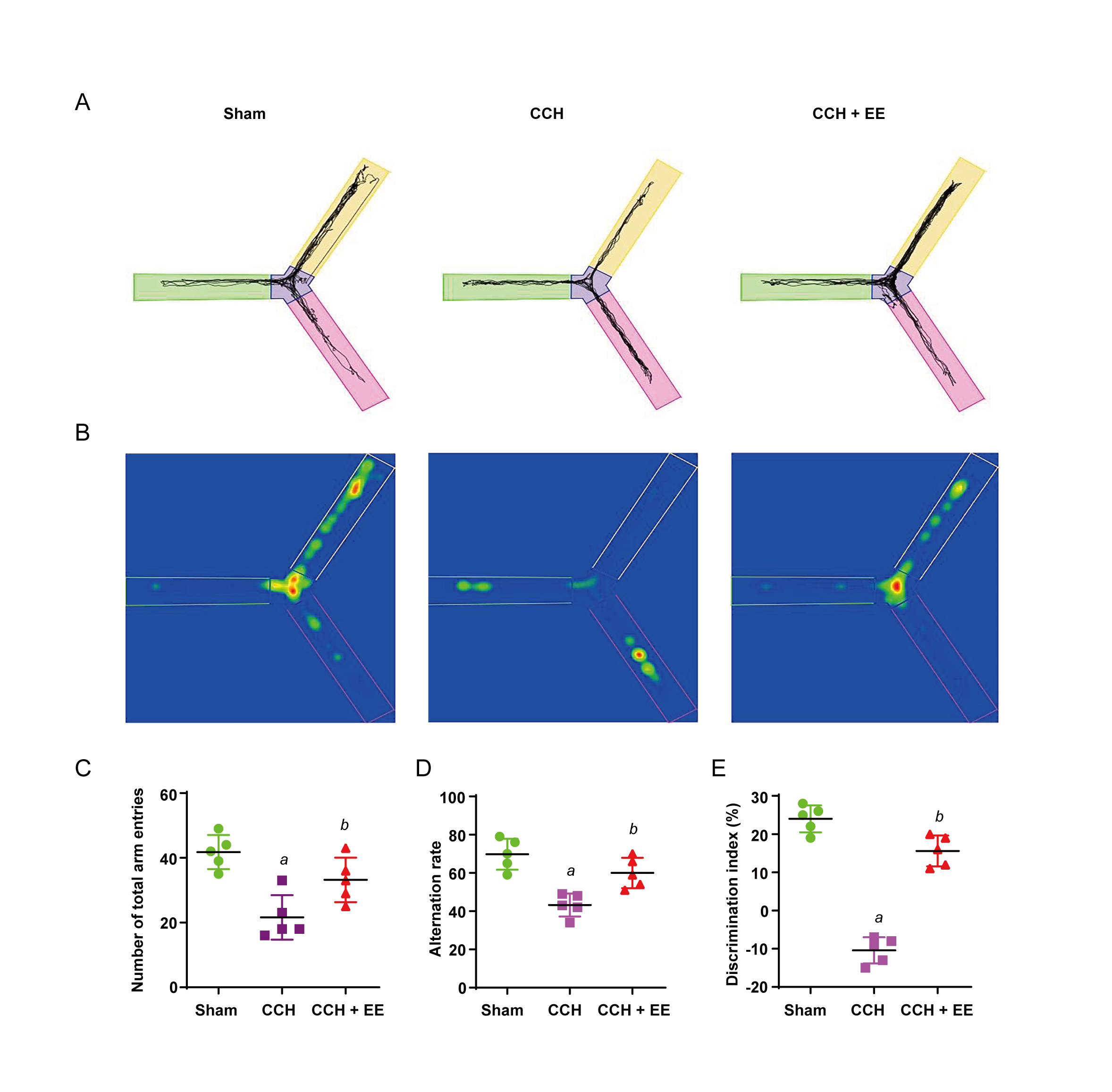

Supplement: Supplementary Figure 1 — Results of Y maze test and NOR test. (A) Movement track of mice. (B) Heat map of mouse movement. (C, D) Behavior analysis of Y maze test. (E) Behavior analysis of NOR test. Values represent the mean ± s.d. aP < 0.05 vs. Sham; bP < 0.05 vs. CCH group (n = 6). [file Image_1.tif]

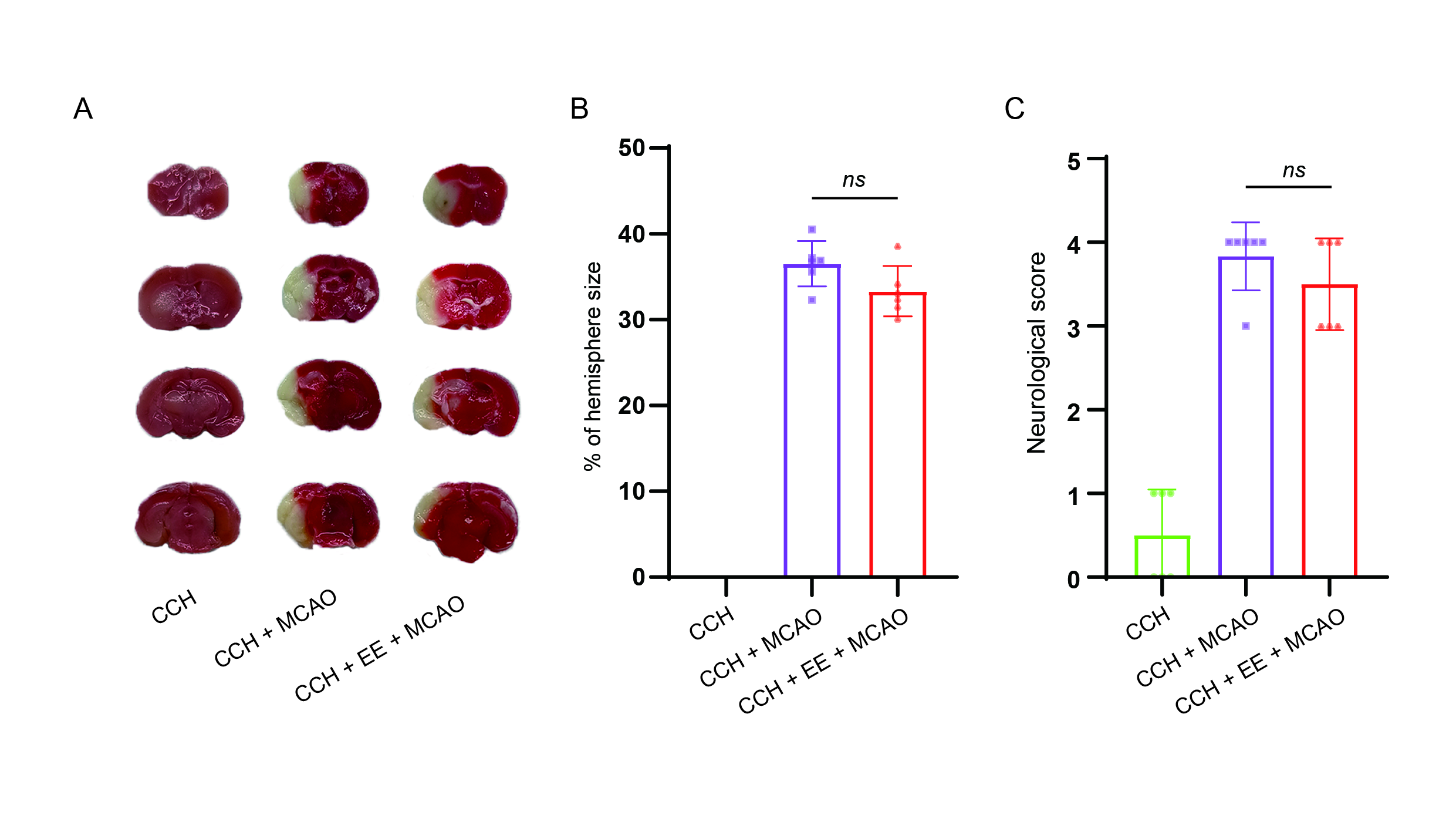

Supplement: Supplementary Figure 2 — Results of TTC staining and neurological score one day after MCAO. (A, B) TTC staining of CCH mice one day after MCAO. (C) Neurological score of CCH mice one day after MCAO. Values represent the mean ± s.d. ns non significance. (n = 6). [file Image_2.tif]

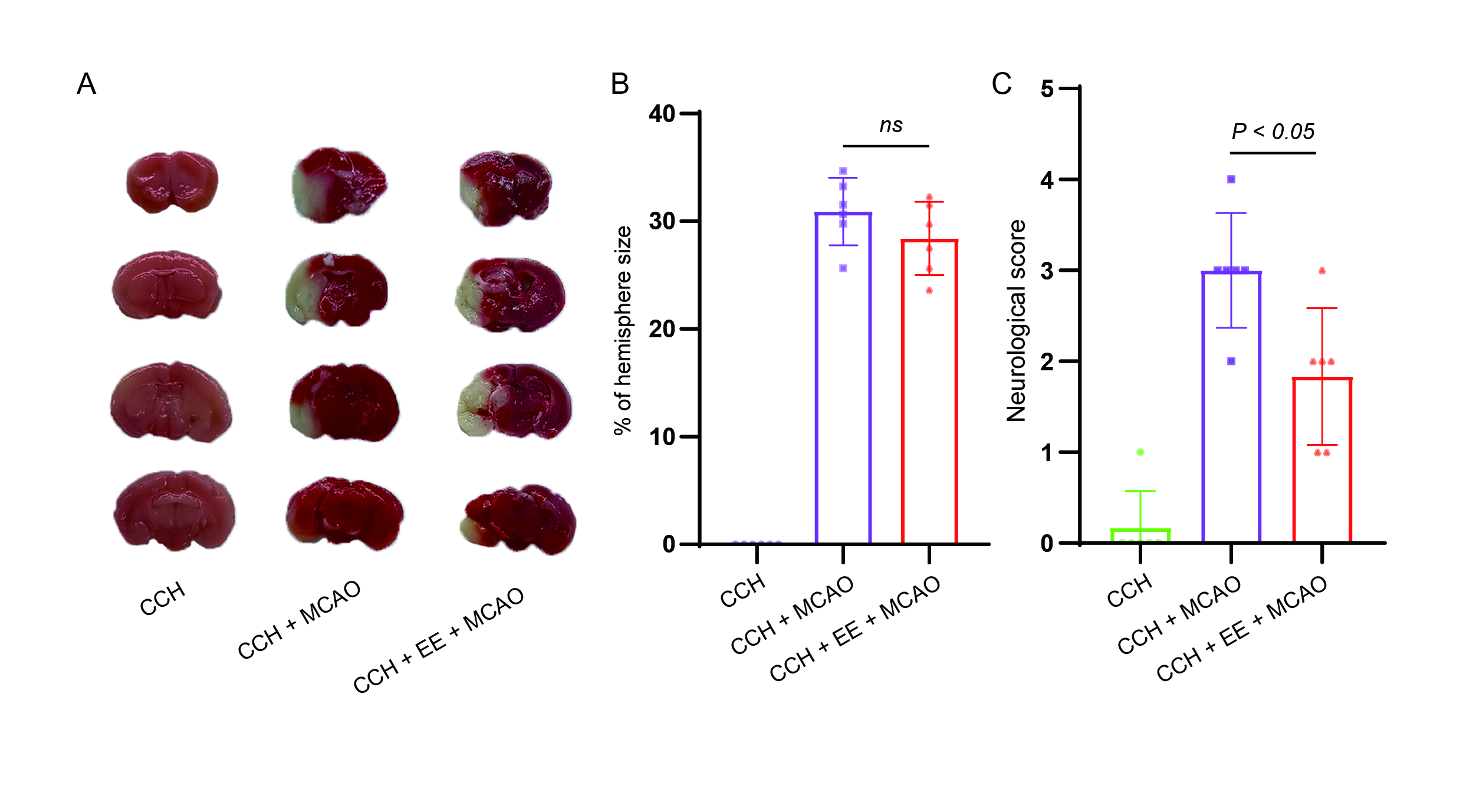

Supplement: Supplementary Figure 3 — Results of TTC staining and neurological score 21 days after MCAO. (A, B) TTC staining of CCH mice 21 days after MCAO. (C) Neurological score of CCH mice 21 days after MCAO. Values represent the mean ± s.d. ns non significance. (n = 6). [file Image_3.tif]
